# Understanding Physical Activity Demands and Reported Perceptions of Fatigue in Children with Developmental Disabilities

**DOI:** 10.3390/bs16060945

**Published:** 2026-06-09

**Authors:** Kavya Iyer, Richard Stevenson, Vydia Permashwar, Brittany Howell, Stephanie DeLuca

**Affiliations:** 1 Graduate Program in Translational Biology, Medicine, and Health, Virginia Tech, Blacksburg, VA 24060, USA; 2 Department of Developmental and Behavioral Pediatrics, University of Virginia, Charlottesville, VA 22908, USA; rds8z@uvahealth.org; 3 Department of General Pediatrics, Carilion Clinic, Roanoke, VA 24018, USA; vpermashwar@carilionclinic.org; 4 Department of Human Development and Family Science, Virginia Tech, Blacksburg, VA 24060, USA; brhowell@vtc.vt.edu; 5 Neuromotor Research Clinic, Fralin Biomedical Research Institute at Virginia Tech Carilion, Roanoke, VA 24016, USA; stephdeluca@vtc.vt.edu

**Keywords:** neuromotor disabilities, physical activity, fatigue, Cerebral Palsy, Down Syndrome, children

## Abstract

Children with developmental disabilities are less physically active and at increased risk for chronic conditions that physical activity might ameliorate. This study examined the relationships between the impact of physical activity (e.g., muscles burning, body tiredness), physical activity time and fatigue in children with Cerebral Palsy (CP) and Down Syndrome (DS) in comparison to typically developing (TD) children. A convenience sample of children and parents was enrolled. All children were between 4 and 10 years, ambulatory, medically stable, not taking sleep-aid medications and were either TD (n = 20) or diagnosed with CP (n = 14) or DS (n = 5). Children and parents separately answered questionnaires about participation in physical activity and fatigue during the past week, yielding retrospective data. Additionally, they completed prospective questionnaires for 3 consecutive days. Repeated measures multivariate and univariate analyses (post hoc) of variance, along with correlations between variables were completed. Analyses of retrospective data yielded no specific findings. Prospectively, all groups of parents reported that as the impact of physical activity increased, perceptions of fatigue decreased (r = −0.349; *p* < 0.001). Parents of children with Cerebral Palsy noted a negative relationship between the time spent doing physical activity and perceptions of fatigue (r = −0.553; *p* < 0.001). Between-group differences in perceptions of fatigue occurred for children with CP compared to TD (F = 8.248; *p* < 0.001). Parent-reported findings suggest potential associations between physical activity participation and perceptions of fatigue across diagnostic groups.

## 1. Introduction

The American Academy of Pediatrics suggests that there has been an increase in the number of developmental disorder/disability diagnoses over the past several years (Durkin, 2019). With this recognition, there is a need to focus on ways to improve long-term health and wellbeing across the lifespan of individuals with developmental disabilities. Many studies acknowledge the impact of comorbid chronic conditions on the short- and long-term health of patients with these diagnoses (Peterson et al., 2020; Dewey, 2018), and increasingly, there are concerns that a lack of healthy lifestyle choices (such as preventative health behaviors including but not encompassing incorporating daily physical activity in the routine, consuming nutrient-dense food, and ensuring healthy sleep patterns/routines) may impact the development or presentation of many of these secondary chronic medical conditions that are separate from/unrelated to the primary disability diagnosis (Kim & Jeon, 2025; Olsen et al., 2021). This has caused there to be a call for greater research into the potential impact of healthy lifestyles choices, such as participation in physical activity.

There is almost unanimous agreement among most professionals that increased physical activity and less-sedentary behaviors positively impact short- and long-term health in almost all populations, including those with developmental disabilities (Olsen et al., 2021; Stancliffe & Anderson, 2017; Brazendale et al., 2020). That said, many studies suggest that children and adults with developmental disabilities are more sedentary than the general population (Hsieh et al., 2017; Leser et al., 2017; Verschuren et al., 2016). This increased sedentary behavior is thought to contribute to the higher risks for chronic comorbidities in both children and adults. Examples of comorbid diagnoses thought to be impacted by less physical activity include cardiovascular disease (Murthy & Hsieh, 2021), chronic pain (Noyek et al., 2024), and sleep apnea (Korb et al., 2023), all of which occur at higher rates in individuals with disabilities in comparison to the general population (Juhasz & Byers, 2025).

The American Heart Association has put forward lifestyle choice recommendations for individuals across the lifespan. ‘Life’s Essential 8’ is an easy-to-understand series of physical activity guidelines for both adults and children (Kumar et al., 2023). These guidelines are put forward to lower the risk of developing chronic conditions such as cardiovascular disease (Sebastian et al., 2025). Adopting suggestions from Life’s Essential 8 such as monitoring weight status, maintaining sustainable and enjoyable physical activity opportunities, consuming well-balanced meals, and monitoring development and progression of cardiovascular diseases by maintaining contact with medical providers is crucial (Strandberg et al., 2025; Masmoum et al., 2024; Feng et al., 2026; Vaidya et al., 2011). Despite these recommendations and the literature supporting the positive benefits of lifestyle choices on long-term health in many varied populations of adults and children, there is little evidence available to confirm or deny the potential impact of these choices on children diagnosed with developmental disabilities. With regard to physical activity recommendations, this may be due in part to the fact that we do not fully understand exactly why such populations are more sedentary, as developmental disabilities all present differently for every child, with varying implications that may preclude participation in physical activity.

Cerebral Palsy and Down Syndrome are two such developmental disorders that are typically diagnosed early in development and have documented reductions in levels of physical activity in comparison to the general population (Lee et al., 2023; Pitetti et al., 2013). Cerebral Palsy is considered a neuromotor developmental disorder that involves static brain lesions. Children experience movement challenges that can be unilateral or bilateral and can include other challenges across developmental domains (Patel et al., 2020). Down Syndrome is a genetic disorder caused by an extra total or partial copy of Chromosome 21, often diagnosed in utero. Children with Down Syndrome present with many different phenotypes, but traditionally may experience global developmental delay and face acute or chronic medical complications with several organ systems and have motor impairments usually associated with hypotonia. For both disorders, levels of sedentary behavior are high (Claassen et al., 2011; Aljuhani et al., 2025), and the known reductions in physical activity are often associated with motor impairments (Graf et al., 2023).

For children with Cerebral Palsy, the 5-year risk of developing cerebrovascular disease, chronic obstructive pulmonary disease, or kidney disease are markedly higher than those who are typically developing (Whitney, 2022). For those with Down Syndrome, adults have an increased chance of developing sleep disorders, respiratory diseases, diabetes, endocrine disorders, obesity, and autoimmune disorders, amongst many others, compared to those who are typically developing (Baksh et al., 2023). These comorbid diagnoses can be debilitating to the patient and their caregivers and may contribute to increased functional decline (Chicoine et al., 2021). However, these diagnoses are also shown to be positively impacted by increased levels of physical activity in other populations (Fu et al., 2025). For example, incorporating healthy lifestyle habits has been shown to slow the onset or progression of several chronic conditions such as hypertension, atherosclerosis, diabetes, and even cancer (Oster & Chaves, 2023; Rahelić et al., 2024).

In 2020, two major organizations focused on Down Syndrome advocacy and clinical care developed research recommendations about the need to focus on chronic and comorbid medical conditions associated with diagnosis (Startin et al., 2020; Hendrix et al., 2021; Diaz, 2020). These organizations acknowledge the impact of chronic comorbidities on the acute and chronic health and wellbeing of patients across different types of disabilities. Additionally, they suggested that the incorporation of healthy lifestyle choices in a patient’s everyday activities may positively impact the development or presentation of many secondary chronic conditions.

Most previous research studies on physical activity in these populations have focused on musculoskeletal limitations and/or the lack of accessible options for physical activity as the primary factors that prevent participation, but less research has been completed on factors that encourage or preclude individuals with developmental disabilities from making positive lifestyle choices (specifically participating in physical activity). Across a number of disability diagnoses, there are varying factors that impact participation in physical activity, including a lack of resources or adaptable modalities, challenges with affording or otherwise accessing resources, and lack of training in how to participate in these activities (Rimmer et al., 2004; Ascondo et al., 2023). Even if the full amount of recommended physical activity is not attained, some weekly physical activity still shows positive benefits (Martin Ginis et al., 2021) when accessible options are available. Researchers have proposed a framework to capture the complexities that influence physical activity participation in children diagnosed with Cerebral Palsy (Duff et al., 2023). The authors theorize that participation in physical activity and motivation to make healthier lifestyle choices is due to internal forces. In this proposal, we contend that this framework and motivational influences are applicable to children across diagnostic categories. One internal driving force that could promote or preclude participation in physical activity may, in fact, be self-perceived levels of fatigue.

Fatigue is a construct that has many proposed definitions based on context, which makes it challenging to operationalize, but it is generally recognized as a ‘feeling’ or ‘perception’ of mental or physical tiredness or exhaustion (Phillips, 2015; Chaudhuri & Behan, 2004). Fatigue has shown to be related to physical activity participation in certain populations, but levels of fatigue in individuals with developmental disabilities are poorly understood, despite chronic fatigue being documented as a symptom across many neurological disorders (Maestri et al., 2020). Due to its subjective nature and variability between every person, and even on a daily basis, elucidating a standard definition of fatigue can certainly be challenging. Amongst the pediatric disability populations, fatigue is particularly important to investigate as it not only can impact the child’s quality of life, but also, separately, may impact the parent’s quality of life as well.

For children with physical and cognitive congenital and acquired disabilities specifically, fatigue has been shown to reduce quality of life and is oftentimes coupled with numerous challenges associated with respective diagnoses (Sokol et al., 2025; Choong et al., 2024; Roma et al., 2019; MacAllister et al., 2009). While there is a paucity of literature available about the relationships between physical activity and fatigue in childhood disability populations, authors have demonstrated that amongst pediatric populations broadly, certain age categories and genders tend to experience greater fatigue with varying levels of physical activity compared to others (Bricout et al., 2025). However, interestingly, authors argue that prepubertal children have metabolic response profiles that are comparable to adult endurance athletes, suggesting that amongst the pediatric population, for those without developmental disabilities, there is an untapped opportunity to understand comparative perceptions of fatigue due to physical activity participation between these populations (Birat et al., 2018).

In this study, the aim is to understand the relationships between physical activity participation and perceptions of fatigue for children diagnosed with Cerebral Palsy and Down Syndrome, two commonly diagnosed developmental disabilities, from both parent and child perspectives. By utilizing this cross-informant approach, we hope to gather information from both parties while still recognizing that children are likely able to report internal behaviors and thoughts better than parents (Caqueo-Urízar et al., 2022). As one of the first studies understanding these constructs in this population, a better understanding of the potential relationships between the constructs of physical activity and fatigue may help clinical communities with actionable targets that can be utilized towards helping individuals with these diagnoses make improved lifestyle choices.

## 2. Materials and Methods

**Figure 1:**
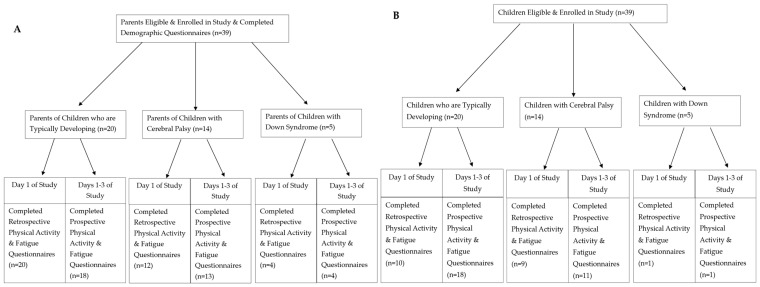
Study flow procedural framework. Study flow diagrams for parent (Panel **A**) and child (Panel **B**) recruitment and questionnaire completion throughout study.

Study Design: This study had a retrospective and prospective observational repeated-measures questionnaire study design. This study was approved by the Virginia Polytechnic Institute and State University Institutional Review Board. Recruitment efforts aimed to enroll both parents/guardians and their children for this study. Enrollment for this study occurred from January 2025 to November 2025. There were two types of questionnaires designed—retrospective and prospective. The former questionnaires were given at the time of enrollment (on Day 1) and queried both parents and children separately about physical activity and fatigue impacts from the previous 7 days (a total of 3 questionnaires each that were completed once). The latter questionnaires were designed to gather data from both parents and children separately about daily physical activity and fatigue across 3 consecutive days (Figure 1; Days 1–3), which is further described in Figure 1. In total, for the prospective questionnaires, parents and children completed 3 separate questionnaires each daily across the 3 days.

Study Location, Participant Recruitment Strategy, and Sampling Approach: Parents provided informed consent, and enrolled children provided assent for participation. Children with no known diagnoses (typically developing) and those with Cerebral Palsy and Down Syndrome were recruited. Recruitment occurred through local, regional, and state agencies, but was open to enrollment across the United States. Research flyers were distributed at outreach events and posted to social media. Specific inclusion/exclusion criteria are listed in Table 1. Eligible families (both parents and children) were recruited using convenience-based sampling.

Study Period: After initial screening eligibility for participating in the study as well as official study enrollment, parents and children participated for a total of 3 consecutive days across 30 days. The specific days were chosen by the family. During this period, parents and children completed tasks that were outlined during the eligibility screening discussion.

Sample Size Calculation: There is limited information with regard to fatigue in children with developmental disabilities within the literature and no studies we could identify compared fatigue in this age group and across these diagnostic categories to justify sample size calculations. Instead we based sample size on reported levels of light physical activity between children with Cerebral Palsy and those who were typically developing (Ryan et al., 2015). Calculations indicated that 20 children per group were needed to detect groups differences with 80% power. To further support sample size calculations, we anticipated a moderate correlation (r > 0.55–0.65) between perceived levels of fatigue and reported levels of impact due to physical activity. At time of study design, we did not hypothesize any directionality of such correlation because of the limited literature surrounding both variables. With a correlation coefficient (r) = 0.65 and a β = 0.20, a sample size of 16 is estimated. R = 0.55 resulted in a needed sample size. Thus, we anticipated that 4 families per group would not complete data collection, thus allowing for 20% study attrition and therefore initially planned for final data analysis to be based on 20 children per group (Teresi et al., 2022).

Data Collection Tools: All questionnaires were designed through the QuestionPro software licensed through Virginia Polytechnic Institute and State University, and completed remotely. All study questionnaires utilized during the study were delivered to families via secure web-based links. Links to these surveys were included on personalized instruction sheets.

Study Questionnaire Adaptation: Questionnaires addressing pediatric physical activity participation impact and fatigue were adapted from the U.S. Department of Health and Human Services Patient Reported Outcomes Measurement Information System (PROMIS) validated questionnaires. The original PROMIS questionnaire for pediatric fatigue for children utilized a version of a Likert scale (never, almost never, sometimes, often, or always) and asked children over the past 7 days if they felt some version of fatigue. The original PROMIS questionnaire for pediatric fatigue for proxies (parents) utilized the same queries and formatting (except it was altered so that parents could provide their inputs). The original PROMIS questionnaire for pediatric physical activity asked children the number of days over the past 7 days that they felt some form of impact due to physical activity. Children were allowed to select between various ranges of days. The original PROMIS questionnaire for pediatric physical activity for proxies (parents) utilized the same general queries and formatting (except it was altered so that parents could provide their inputs).

Adaptations were made because original PROMIS questionnaires were designed to assess perspectives of typically developing children and include modifications that sought to be more inclusive of children diagnosed with developmental limitations. We additionally adapted these questionnaires so that they could serve as both retrospective and prospective tools. The topics for the physical activity questionnaires from the original to the adapted versions were identical. The topics of the fatigue questionnaires from the original to the adapted were condensed and combined to a total of 4 questions per questionnaire instead of the original 10.

There were questionnaires that asked parents and children about physical activity and fatigue retrospectively (i.e., during the last 7 days), and questionnaires designed to gather data prospectively across 3 days. Specific topics addressed for each questionnaire are shown in Table 2. Parents and children either affirmatively or negatively responded to each question each for the prospective questionnaires on fatigue and the impact of physical activity. The number of ‘Yes’ responses thus created a total score. The retrospective surveys asked about the previous 7 days and were completed at the time of enrollment. The prospective surveys were completed for 3 consecutive days that were chosen by parents. Parents and children also reported on amounts of time that the enrolled child completed physical activity for the same periods.

Specific questionnaires used in this study can be found in the Supplemental Material. The demographic questionnaire (see Questionnaire S1) was utilized to gather age, race, ethnicity, sex, and diagnostic information. The physical activity retrospective questionnaires were provided to both parents (see Questionnaire S2) and children (see Questionnaire S3) to assess the child’s physical activity impact over the 7 days prior to commencement of the study. The prospective physical activity questionnaires were provided to both parents (see Questionnaire S4) and children (see Questionnaire S5) to assess the child’s physical activity impacts during the 3 consecutive days of the study. The fatigue retrospective questionnaires were provided to both parents (see Questionnaire S6) and children (see Questionnaire S7) to assess the child’s impact due to fatigue over the 7 days prior to commencement of the study. The prospective fatigue questionnaires were provided to both parents (see Questionnaire S8) and children (see Questionnaire S9) to assess the child’s impact due to daily fatigue during the 3 consecutive days of the study.

Data Analysis. Data analyses included descriptive characteristics of all variables. Correlations between variables across and between groups were completed. For data documenting retrospective information (last 7 days), multivariate analyses of variance were completed, and followed by univariate analysis of variance using least squared differences for post hoc analyses to delineate group differences. For data collected prospectively, repeated measures multivariate analyses of variance were completed, and followed by univariate analysis of variance using least squared differences for post hoc analyses to delineate group differences.

## 3. Results

Table 3 provides demographic and participant information for the 39 enrolled children and families. Of note, parents indicated that most enrolled children with Down Syndrome were unable to complete child-specific questionnaires. This is discussed in the study limitations.

### 3.1. Retrospective Analyses

Parent Responses: There were no significant correlations between physical activity impact and perceptions of fatigue as well as time spent on physical activity and perceptions of fatigue respectively across diagnoses based off parental retrospective reports. Parents of children specifically who were typically developing did retrospectively report an inverse relationship between the time spent doing physical activity and fatigue (r = −0.487; *p* = 0.029). There were no significant correlations between physical activity impact and fatigue separated by diagnoses. Between-group differences in these factors based off of parental reports were also not observed. Table 4 below describes the mean number of days over the past week that parents believed their child faced physical activity impact and perceptions of fatigue, as well as the daily number of minutes doing physical activity over the past week.

Child Responses: There were no significant correlations between physical activity impact and perceptions of fatigue as well as time spent on physical activity and perceptions of fatigue respectively across diagnoses based off of the children’s retrospective reports. There were no significant correlations between physical activity impact, time spent doing physical activity, and fatigue separated by diagnoses. Between-group differences in these factors based off of the children’s reports were also not observed. Table 4 above describes the mean number of days over the past week that the child faced physical activity impacts and perceptions of fatigue, as well as the daily number of minutes doing physical activity over the past week based on parental perspective. Of note, due to limited response counts for children with Down Syndrome (n = 1), associated descriptive statistics are not reported.

### 3.2. Prospective Analyses

Parent Responses: Parents did report a significant inverse relationship across diagnoses between physical activity impact and perceptions of fatigue (r = −0.349; *p* < 0.001), supporting that as physical activity impact increased, perceptions of fatigue decreased. Parents of children specifically with Cerebral Palsy reported a negative relationship between physical activity impact and fatigue (r = −0.513; *p* = 0.002). Additionally, parents of children with Cerebral Palsy reported a negative relationship between the time spent doing physical activity and perceptions of fatigue (r = −0.553; *p* < 0.001). Repeated-measures analysis indicated no main effect for day on any variable but indicated a main effect for diagnosis on fatigue (F = 4.944; *p* = 0.019). Table 5 describes the mean number of affirmative responses for each questionnaire based on parental perspectives in all three diagnostic categories. Parents did report an increase in perceptions of fatigue for children with CP compared to TD individuals (F = 8.248; *p* < 0.001).

Child Responses: There were no significant correlations between physical activity impact and perceptions of fatigue as well as time spent on physical activity and perceptions of fatigue respectively across diagnoses based off the children’s prospective reports. Repeated measures analysis yielded no significant findings on any variable. Table 5 above describes the mean number of affirmative responses for each questionnaire based on child perspectives in all three diagnostic categories.

Retrospectively, parent and child reports of the number of minutes spent doing physical activity daily over the prior week were not noticeably different, with a moderately positive correlation of r = 0.383. For children with Cerebral Palsy, parents reported children participating on average 91.67 ± 65.48 min of physical activity per day over the week prior to the study commencement compared to a child-reported average of 97.86 ± 121.24. Parents of children with Down Syndrome reported on average 25.00 ± 15.81 min of physical activity per day over the past 7 days prior to study commencement. Parents of typically developing children reported an average of 69.16 ± 77.18 min of physical activity daily over the prior week compared to children reporting 62.40 ± 55.89 min per day.

Prospectively the parent and children reports of the number of minutes spent doing physical activity did not differ significantly and were moderately positively correlated (r = 0.385). For children with Cerebral Palsy, parents reported an average number of daily minutes of physical activity across the 3 days of 144.37 ± 80.24 compared to the child-reported average of 230.13 ± 172.11. Parents of children with Down Syndrome reported an average number of daily minutes of physical activity of 162.83 ± 3.06; no child reports in this diagnostic group were reported. Parents of typically developing children reported an average number of daily minutes of physical activity across the 3 days of 232.47 ± 234.06 compared to a child-reported average of 165.11 ± 83.68.

## 4. Discussion

This was one of the first studies to our knowledge that utilized retrospective and prospective parental and child perspectives to elucidate current physical activity participation and feelings of fatigue for children with developmental disabilities. By including the parental point of view, we wanted to ensure that we collected information from the ‘first person’s’ point of view of the children, but also from the parents, who oftentimes can understand factors externally that children may not be able to internalize. In this study, parents were able to recognize fatigue in their children, while children could not internalize the factor. This could be for several reasons—including that fatigue is, in general, a challenging variable to contextualize. Children may also have limited insight into the meaning of fatigue and its role in accomplishing daily activities. While there were no group differences in perceptions of fatigue amongst children, parents with children with Cerebral Palsy reported increased levels of child fatigue compared to parents with children who were typically developing. This mirrors the existing literature that also suggests that adolescents with Cerebral Palsy face greater impacts due to fatigue compared to age-matched adolescents who were typically developing (Brunton et al., 2021), but beyond this, there is very little information addressing the impact of fatigue on children with Cerebral Palsy. It is widely understood, however, that individuals broadly across all ages with Cerebral Palsy oftentimes face chronic fatigue. There is also a paucity of literature utilizing cross-informant data to assess these factors. Also, since parents are likely to know the child’s day-to-day patterns, they would be able to recognize any deviations from the norm. We also potentially believe that the child’s response could have been driven from the instantaneous and immediate feeling of fatigue while completing the questionnaire, and its perception could of course vary throughout the day, and it could be challenging to reflect on past experiences of fatigue.

In prospective analyses, parents did report higher levels of fatigue in children with Cerebral Palsy compared to those who were typically developing, despite the fact that the impact due to physical activity and time spent on daily physical activity remained the same. We assume that this may be due to the overall increased effort or possible energy expenditure that those with disabilities tend to face. Additionally, while this certainly depends on the individual, there is also the potential for diagnostic variability in the physiological impact of physical activity. Alongside the belief that fatigue played a role in the time or type of physical activity participation in children with disabilities, authors also suggest that a lack of available physical activity opportunities that were safe and accessible for those with disabilities was prevalent, along with low self-efficacy and a lack of trained staff, guidance, and support in educational settings (MacEachern et al., 2021; Yu et al., 2022; Liu et al., 2025; Shields & Synnot, 2016).

From both parental and child perspectives, we did not find any retrospective group differences. While this could be for several factors, including indeed the fact that children did not feel fatigue due to physical activity participation, we also believe this could be due to potential recall bias by all parties. However, parents of children who were typically developing did report an inverse relationship between time spent on physical activity and perceived fatigue. Prospectively, parents reported a significant inverse relationship between physical activity and fatigue—as the impact due to physical activity increases, perceptions of fatigue decrease across diagnoses. This could suggest that if a child faces a higher amount of fatigue, they may avoid doing further physical activity as a protective mechanism to prevent further fatigue. Alternatively, as physical activity increases, children may see more physiological benefits that could condition them to perform better, thus lowering the chances for future fatigue. In broader exercise metabolism and medicine research, authors do suggest that adopting moderate to vigorous physical activity regimens may in turn reduce overall perceptions of fatigue (Wender et al., 2022; Puetz, 2006) and aid as a ‘prescription’ for those facing fatigue due to preexisting acute or chronic conditions (Barz et al., 2024). For medically vulnerable populations such as the elderly, authors further emphasize the need for engaging and comprehensive physical activity opportunities, as research has conversely shown that as physical activity decreases, reported perceptions of fatigue increase (Egerton et al., 2016).

Organizations such as the American Academy of Pediatrics as well as others should continue to recommend physical activity as a health prevention metric for children. However, care should be made to consider adaptations to recommendations for children with disabilities, as the amount and type of physical activity can vary based on diagnosis and age. Additionally, advocacy powerhouses should continue to prioritize the need for equitable access to opportunities to engage in healthy lifestyles through child-friendly adaptive physical activities and sports. Ensuring inclusivity is essential to promoting long-term mental and physical wellbeing. Lastly, we believe that further work needs to be done by clinical teams to understand the role of fatigue in the overall daily quality of life for children with disabilities.

### Limitations

One of the most apparent limitations of this study was the uneven sample size distribution, particularly for families of children with Down Syndrome. Our hope is to increase targeted and proactive recruitment efforts to obtain larger sample sizes for future studies. Given that we had both parents and children complete retrospective and prospective questionnaires, we did realize that we were unable to get the perspectives of children with Down Syndrome due to disclosed comprehension barriers. For future studies, we hope to include visual or picture-based options in the questionnaires for children with these barriers to further engage with them. While this study did provide unique viewpoints from multiple perspectives, we were not able to corroborate physical activity reports with intensity. It may be beneficial to utilize pediatric accelerometers to capture movement and heart rate data. Lastly, our goal for future studies is to increase the monitoring period of prospective analyses to about 7 days instead of 3 consecutive days. The three-day window may not have been enough time to provide a comprehensive outlook into the potential daily variability in the children’s physical activity and fatigue patterns.

## 5. Conclusions

Parents of children with disabilities reported a significant increase in fatigue compared to those who were typically developing. Parents of children who were typically developing did report an inverse relationship between time spent on physical activity and fatigue, and across diagnoses, parents reported inverse relationships between physical activity impact and fatigue. These findings are particularly novel, as the pediatric disability literature that is specifically focused on lifestyle improvement and lifestyle medicine is particularly limited. Through both retrospective and prospective queries, we found that children faced increased challenges with identifying the concept of fatigue, with potential explanations aligning with questionnaire limitations, developmental comprehension variations, or response burden. Overall, these topics are certainly multifaceted and complex and certainly require further studies to optimize pediatric growth and development. This is the first of what we hope are several studies to come that investigate the role of physical activity on fatigue and overall quality of life for children with disabilities.

## Figures and Tables

**Table 1:** Study inclusion/exclusion criteria.

Inclusion	Exclusion
Must be diagnosed with Cerebral Palsy, Down Syndrome, or be Typically Developing	Is not diagnosed with Cerebral Palsy, Down Syndrome, or be Typically Developing
Must be aged 4–10 years old	Below 4 years or above 10 years of age
Ambulatory Status	Non-Ambulatory Status
Medically Stable	Medically Fragile
Not Taking Sleep-Aid Medications	Taking Sleep-Aid Medications

**Table 2:** Topics addressed in study questionnaires.

Topic Area	Number of Questions	Question Themes
Physical Activity Impact	7	Body tiredness; Length of challenging exercise; breathing hard; Sweating; Muscles burning; Feeling tired; Length of activity
Perceptions of Fatigue	4	Starting and completing activities; Schoolwork; Enjoyment; Paying attention
Physical Activity Participation	1	Approximate number of minutes spent on daily physical activity

**Table 3:** Baseline characteristics of enrolled families by diagnosis.

N = 39		Typically Developing (n = 20)	Cerebral Palsy (n = 14)	Down Syndrome (n = 5)
Child’s Mean Age in Years (S.D.) Upon Study Commencement		7.07 (2.04)	6.71 (1.98)	6.60 (1.52)
Child Race (parent-reported)	White	11	9	5
	Black/African American	2	0	0
	American Indian or Pacific Islander	0	1	0
	Asian	4	1	0
	More than One Race	3	3	0
	Other	0	0	0
Child Ethnicity (parent-reported)	Hispanic	2	2	0
	Non-Hispanic	18	12	5
	Not Reported	0	0	0
Child Sex	Male	7	8	2
	Female	13	6	3

**Table 4:** Retrospective responses indicating average number of days over the past week that parents and children reported child physical activity and fatigue constructs.

**Adult**			
	Cerebral Palsy (n = 12)	Down Syndrome (n = 4)	Typically Developing (n = 20)
Physical Activity Impact, days (SD)	3.35 (1.71)	2.18 (0.46)	3.25 (1.64)
Perceptions of Fatigue, days (SD)	1.10 (0.86)	1.31 (0.90)	0.91 (0.94)
Minutes Spent Daily on Physical Activity (SD)	91.67 (65.48)	25.00 (15.81)	69.16 (77.18)
**Child**			
	Cerebral Palsy (n = 9)		Typically Developing (n = 10)
Physical Activity Impact, days (SD)	3.16 (1.36)		3.14 (1.61)
Perceptions of Fatigue, days (SD)	1.39 (1.46)		0.85 (1.06)
Minutes Spent Daily on Physical Activity (SD)	97.86 (121.24)		62.40 (55.89)

**Table 5:** Prospective responses indicating total average score of responses of parent- and child-reported physical activity questionnaires.

**Adult**			
	Cerebral Palsy (n = 13)	Down Syndrome (n = 4)	Typically Developing (n = 18)
Physical Activity Impact (SD)	3.09 (2.26)	3.14 (1.57)	3.65 (1.97)
Perceptions of Fatigue (SD)	0.82 (1.27)	0.43 (0.54)	0.08 (0.28)
Minutes Spent Daily on Physical Activity (SD)	66.32 (40.73)	65.86 (20.01)	96.90 (129.18)
**Child**			
	Cerebral Palsy (n = 11)		Typically Developing (n = 18)
Physical Activity Impact (SD)	3.41 (1.86)		3.92 (2.09)
Perceptions of Fatigue (SD)	0.63 (0.87)		0.35 (0.84)
Minutes Spent Daily on Physical Activity (SD)	92.00 (86.84)		71.71 (40.10)

## Data Availability

Data is not publicly available. If researchers are interested in obtaining the data, please contact the corresponding author.

## Supplementary Materials

The following supporting information can be downloaded at: https://www.mdpi.com/article/10.3390/bs16060945/s1, Questionnaire S1: Demographics questionnaire; Questionnaire S2: Parent retrospective physical activity questionnaire; Questionnaire S3: Child retrospective physical activity questionnaire; Questionnaire S4: Parent prospective physical activity questionnaire; Questionnaire S5: Child prospective physical activity questionnaire; Questionnaire S6: Parent retrospective fatigue questionnaire; Questionnaire S7: Child retrospective fatigue questionnaire; Questionnaire S8: Parent prospective fatigue questionnaire; Questionnaire S9: Child prospective fatigue questionnaire.
