# Supplementary material for: Understanding Physical Activity Demands and Reported Perceptions of Fatigue in Children with Developmental Disabilities
Source: Behav Sci (Basel). 2026 Jun 9;16(6):945. doi: 10.3390/bs16060945 (PMC13295626; doi:10.3390/bs16060945)
Supplement: Supplementary file 1 [file behavsci-16-00945-s001.zip › behavsci-4289109-supplementary.pdf]

S1- Demographic Questionnaire

Thank you for your participation in our research study (Virginia Tech IRB #: 24-706). Please fill out the below questions asking about basic demographic information for you and diagnostic information for your child. We look forward to working with you and your child! Thank you for contributing to this valuable branch of research.

Study ID #: (Provided by Research Team)

Parent/Guardian Information

Today's Date

Month Day Year

Which state do you live in?

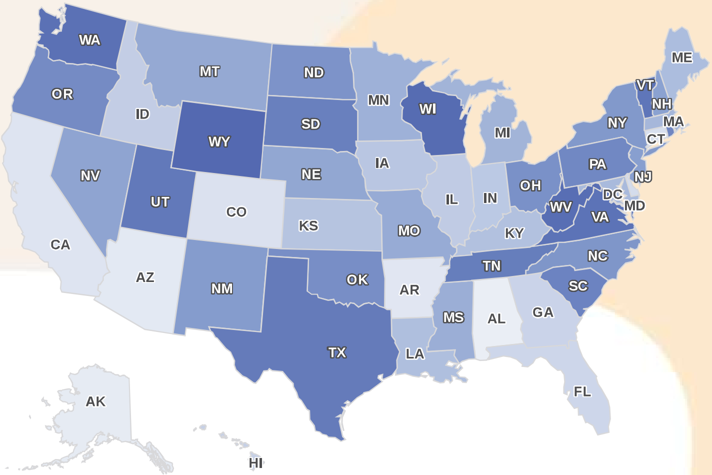

Contact Information for Parent/Guardian #1

First Name

Last Name

Phone

Email Address

Which methods of communication would you prefer that we use to contact Parent/Guardian #1? Please select all that apply.

- ☐ Email
- ☐ Phone
- ☐ Text
- ☐ N/A

Contact Information for Parent/Guardian #2

First Name

Last Name

Phone

Email Address

Which methods of communication would you prefer that we use to contact Parent/Guardian #2? Please select all that apply.

- [ ] Email
- [ ] Phone
- [ ] Text
- [ ] N/A

Child Information

What is your child's month and year of birth? Please ignore the 'day' prompt when inputting this information.

Month Day Year

What is your child's gender?

What is your child's ethnicity?

- [ ] Hispanic or Latino
- [ ] Not Hispanic or Latino

What is your child's race? Please select all that apply.

- [ ] American Indian or Alaska Native
- [ ] Asian
- [ ] Black or African American
- [ ] Native Hawaiian or Pacific Islander
- [ ] White
- [ ] Other

When was your child's most recent medical appointment?

Month Day Year

What is your child's approximate height in inches? (based off of most recent medical appointment)

What is your child's approximate weight in kilograms? (can be based off of most recent medical appointment)

Please list your child's neuromotor disorder diagnosis/diagnoses

Please list your child's secondary medical diagnoses (if applicable)

Approximately how old was your child when they were diagnosed with the neuromotor disability?

Does your child take any medications (either over-the-counter or prescription) regularly for assistance with sleeping?

- ◯ Yes
- ◯ No

Does your child have any food allergies?

- ◯ Yes
- ◯ No

Please provide specifics of what types of foods that your child is allergic to.

Is there any additional information that you would like to provide?

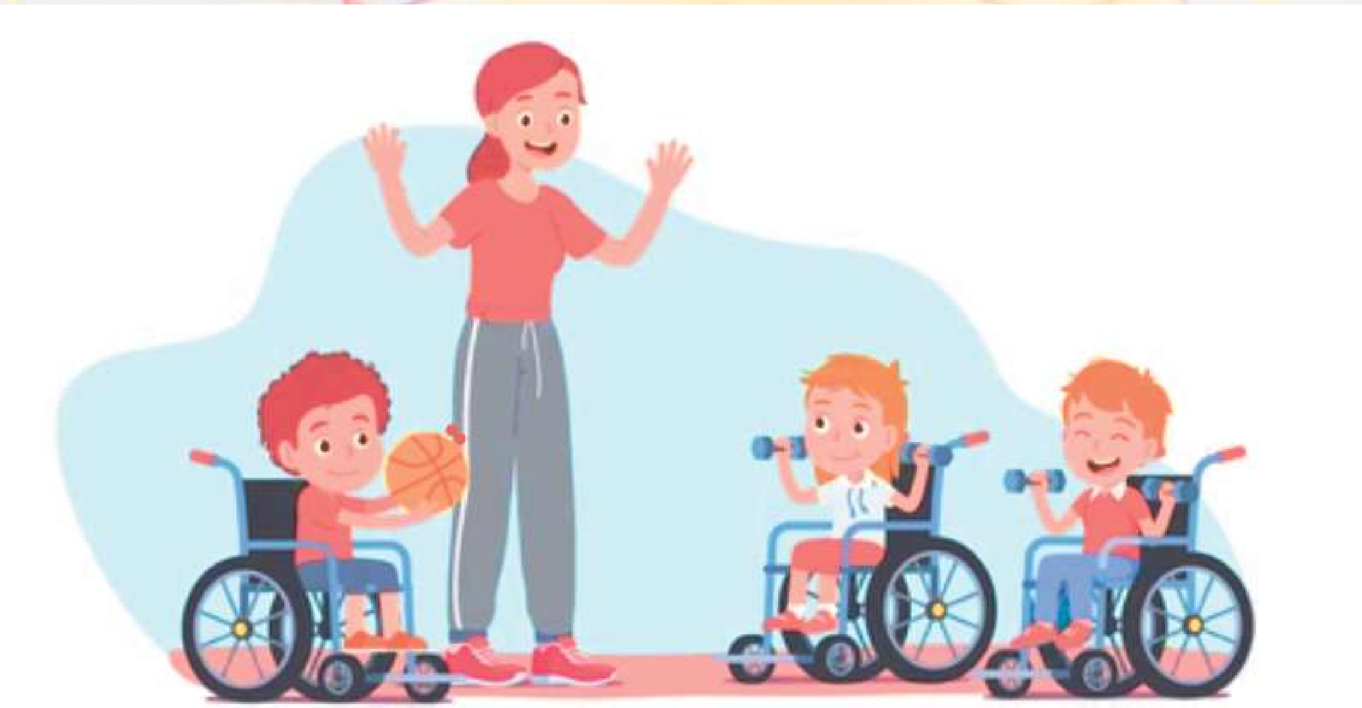

Physical Activity Over the Past Week (Retrospective) (Parent)

QuestionPro

Please think back over the past 7 days and select the best response to the questions below about the number of days that your child felt an impact while exercising

Study ID #: (Provided by Research Team)

Tiredness Over the Past 7 Days

Today's Date

Month | Day | Year

Physical Activity and Body Tiredness

Over the past 7 days, how many days did your child exercise or play so hard that their body got tired?

- 0
- 1
- 2
- 3
- 4
- 5
- 6 or more

Physical Activity and Length of Exercising

Over the past 7 days, how many days did your child exercise really hard for 10 minutes or more?

- 0
- 1
- 2
- 3
- 4
- 5
- 6 or more

Physical Activity and Breathing Hard

Over the past 7 days, how many days did your child exercise so much that they started breathing hard?

- 0
- 1
- 2
- 3
- 4
- 5
- 6 or more

Physical Activity and Sweating

Over the past 7 days, how many days did your child sweat during or after exercising?

- ◯ 0
- ◯ 1
- ◯ 2
- ◯ 3
- ◯ 4
- ◯ 5
- ◯ 6 or more

Physical Activity and Muscles Burning

Over the past 7 days, how many days did your child exercise or play so hard that their muscles burned?

- ◯ 0
- ◯ 1
- ◯ 2
- ◯ 3
- ◯ 4
- ◯ 5
- ◯ 6 or more

QuestionPro

Physical Activity and Feeling Tired

Over the past 7 days, how many days did your child exercise or play so hard that they felt tired?

- 0
- 1
- 2
- 3
- 4
- 5
- 6 or more

Physical Activity and Length of Activity

Over the past 7 days, how many days was your child physically active for 10 minutes or more?

- 0
- 1
- 2
- 3
- 4
- 5
- 6 or more

QuestionPro

Over the past 7 days, on average, how many minutes was your child physically active every day?

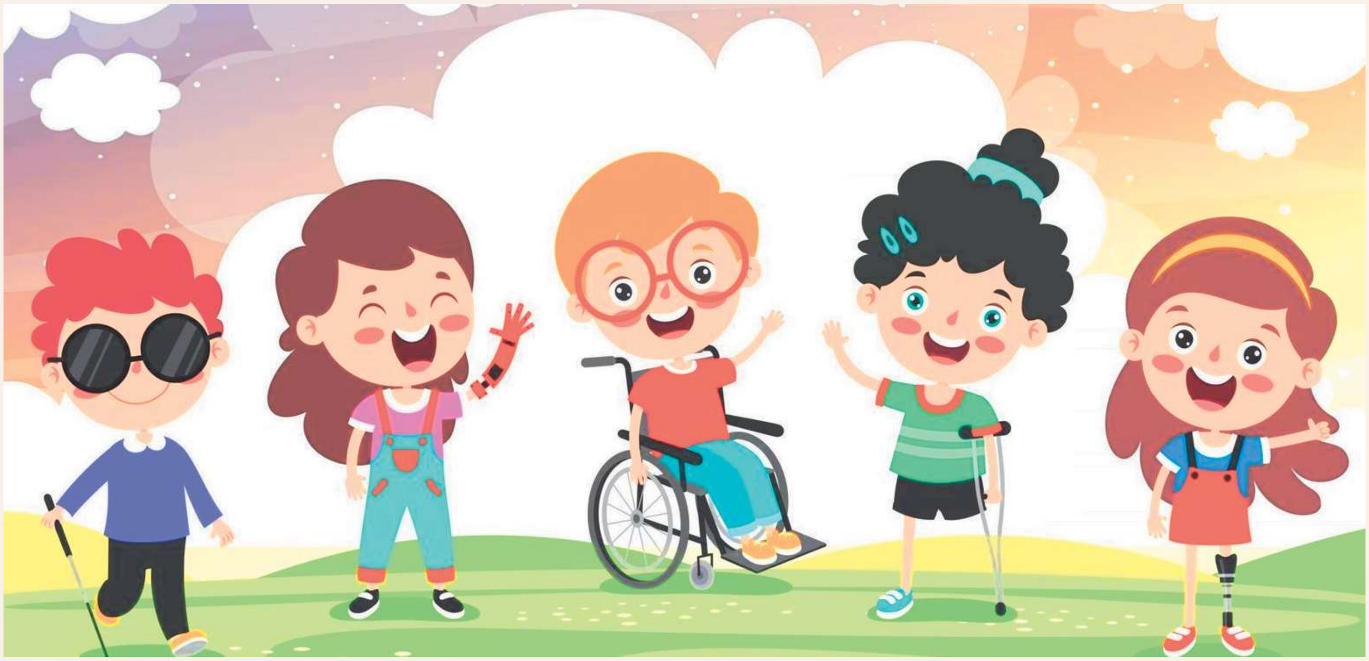

Physical Activity over the Past 7 Days (Child)

Please think back over the past 7 days and select the best response to the questions below about the number of days that you felt a feeling while exercising

Study ID #: (Provided by Research Team)

Tiredness Over the Past 7 Days

Today's Date

Month Day Year

Physical Activity and Body Tiredness

Over the past 7 days, how many days did you exercise or play so hard that your body got tired?

- 0
- 1
- 2
- 3
- 4
- 5
- 6 or more

QuestionPro

Physical Activity and Length of Exercising

Over the past 7 days, how many days did you exercise really hard for 10 minutes or more?

- 0
- 1
- 2
- 3
- 4
- 5
- 6 or more

Physical Activity and Breathing Hard

Over the past 7 days, how many days did you exercise so much that you breathed hard?

- 0
- 1
- 2
- 3
- 4
- 5
- 6 or more

Physical Activity and Sweating

Over the past 7 days, how many days were you so physically active that you sweated?

- 0
- 1
- 2
- 3
- 4
- 5
- 6 or more

Physical Activity and Muscles Burning

Over the past 7 days, how many days did you exercise or play so hard that your muscles burned?

- 0
- 1
- 2
- 3
- 4
- 5
- 6 or more

Physical Activity and Feeling Tired

Over the past 7 days, how many days did you exercise or play so hard that you felt tired?

- ◯ 0
- ◯ 1
- ◯ 2
- ◯ 3
- ◯ 4
- ◯ 5
- ◯ 6 or more

Physical Activity and Length of Activity

Over the past 7 days, how many days were you physically active for 10 minutes or more?

- ◯ 0
- ◯ 1
- ◯ 2
- ◯ 3
- ◯ 4
- ◯ 5
- ◯ 6 or more

Over the past 7 days, about how many minutes were you physically active every day?

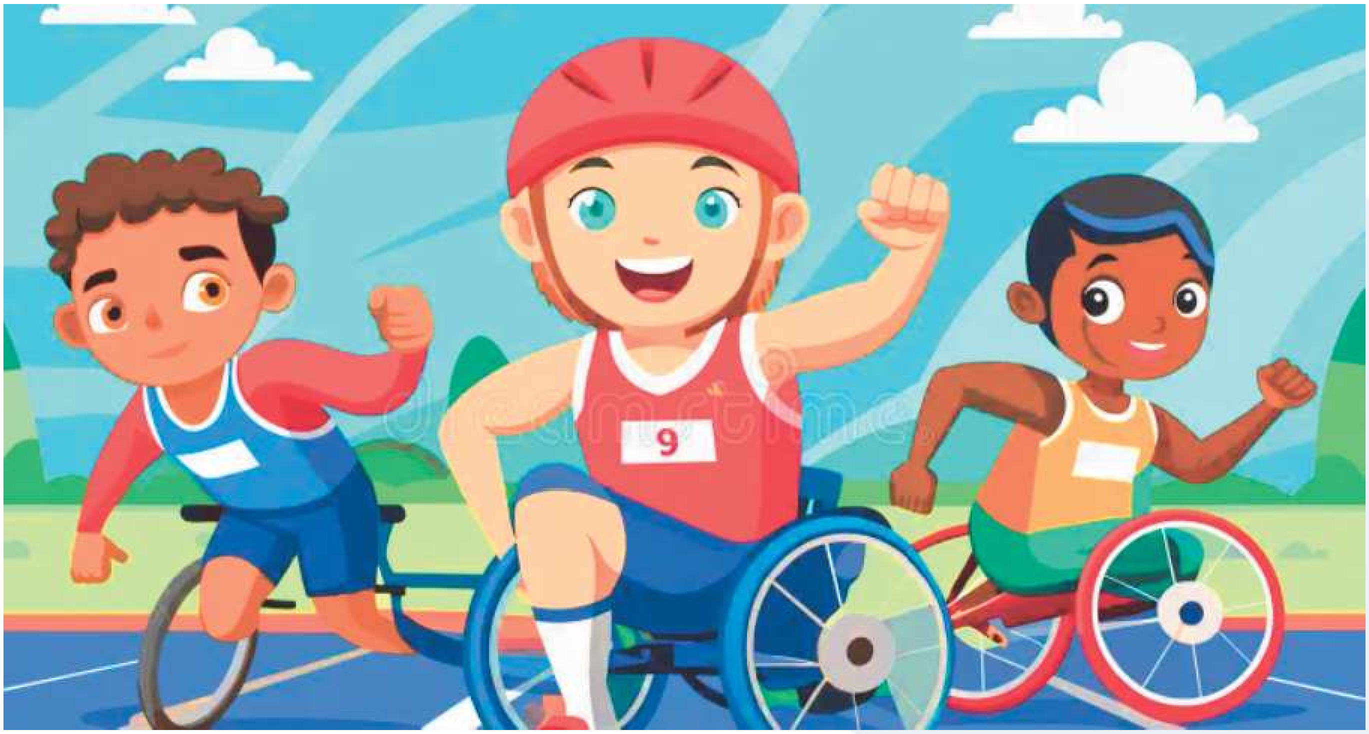

Child's Daily Physical Activity Impact (Parents)

Please select a choice about your child's participation in physical activity/exercise today

Study ID #: (Provided by Research Team)

Tiredness Over the Past 7 Days

Today's Date

Month | Day | Year

Physical Activity and Body Tiredness

Today, did your child exercise or play so hard that their body got tired?

- ◯ Yes
- ◯ No
- ◯ N/A- my child did not exercise or play hard today

Physical Activity and Length of Exercising

Today, did your child exercise really hard for 10 minutes or more?

- ◯ Yes
- ◯ No

- N/A- my child did not exercise really hard today

Physical Activity and Breathing Hard

Today, did your child exercise so much that they breathed hard?

- Yes
- No
- N/A- my child did not exercise today

Physical Activity and Sweating

Today, was your child so physically active that they sweated?

- Yes
- No
- N/A- my child was not physically active

Physical Activity and Muscles Burning

Today, did your child exercise or play so hard that their muscles burned?

- Yes
- No
- N/A- my child did not exercise today

Physical Activity and Feeling Tired

Today, did your child exercise or play so hard that they felt tired?

- ◯ Yes
- ◯ No
- ◯ N/A- my child did not exercise today

Physical Activity and Length of Activity

Today, was your child physically active for 10 minutes or more?

- ◯ Yes
- ◯ No
- ◯ N/A- my child was not physically active today

Today, how many minutes was your child physically active?

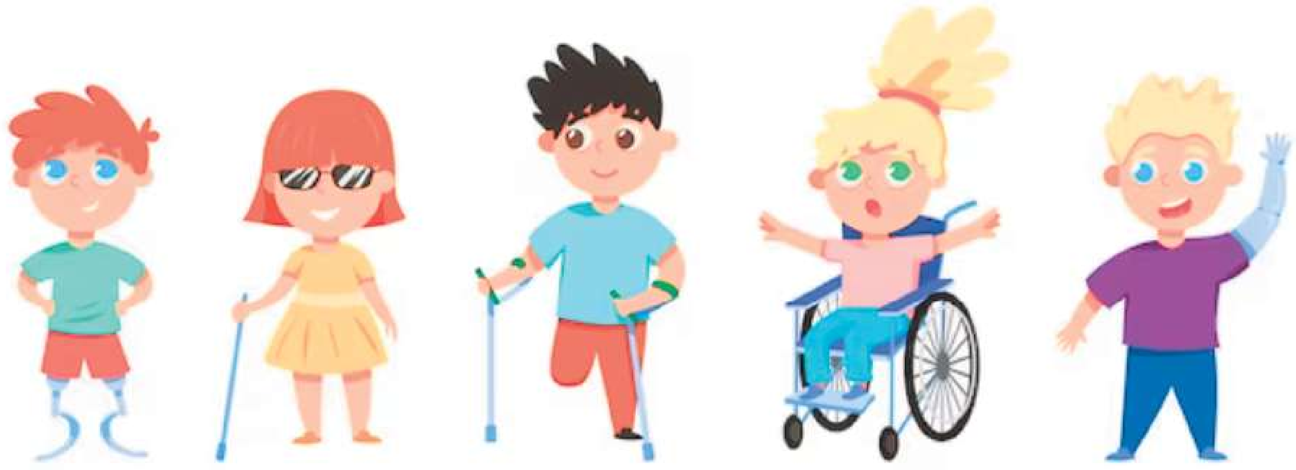

Daily Physical Activity Impact (Child)

Please select a choice about how you felt if you exercised today

Study ID #: (Provided by Research Team)

Tiredness Over the Past 7 Days

Today's Date

Month Day Year

Physical Activity and Body Tiredness

Today, did you exercise or play so hard that your body got tired?

- ◯ Yes
- ◯ No
- ◯ N/A- I did not exercise or play hard today

Physical Activity and Length of Exercising

Today, did you exercise really hard for 10 minutes or more?

- ◯ Yes
- ◯ No

- N/A- I did not exercise really hard today

Physical Activity and Breathing Hard

Today, did you exercise so much that they breathed hard?

- Yes
- No
- N/A- I did not exercise today

Physical Activity and Sweating

Today, were you so physically active that they sweated?

- Yes
- No
- N/A- I was not physically active

Physical Activity and Muscles Burning

Today, did you exercise or play so hard that your muscles burned?

- Yes
- No
- N/A- I did not exercise today

Physical Activity and Feeling Tired

Today, did you exercise or play so hard that they felt tired?

- ◯ Yes
- ◯ No
- ◯ N/A- I did not exercise today

Physical Activity and Length of Activity

Today, were you physically active for 10 minutes or more?

- ◯ Yes
- ◯ No
- ◯ N/A- I was not physically active today

Today, how many minutes were you physically active?

____

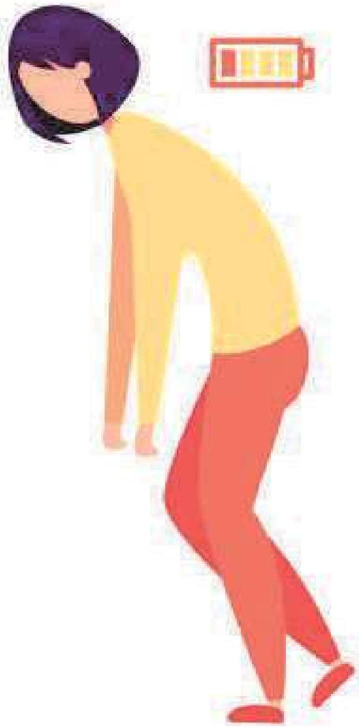

Child's Thoughts of Tiredness over the Past 7 Days (Parent/Guardian)

QuestionPro

Please select a response to the questions below on the number of your days your child was impacted during the day because they were tired

Study ID #: (Provided by Research Team)

Past 7 Days Thoughts on Fatigue

Today's Date

Month Day Year

Fatigue & Enjoyment

Over the past 7 days, if your child appeared tired, how many days was it challenging for them to go outside, play with friends, or do anything they typically enjoy doing as much as they would like?

- 0
- 1
- 2
- 3
- 4
- 5
- 6 or more

Fatigue & Starting & Completing Activities

Over the past 7 days, if your child appeared tired, how many days was it difficult for them to start or complete activities that are normally done daily?

- ◯ 0
- ◯ 1
- ◯ 2
- ◯ 3
- ◯ 4
- ◯ 5
- ◯ 6 or more

Fatigue & Schoolwork

Over the past 7 days, if your child appeared tired, how many days was it difficult for them to keep up with their schoolwork?

- ◯ 0
- ◯ 1
- ◯ 2
- ◯ 3
- ◯ 4
- ◯ 5

- ◯ 6 or more

Fatigue & Paying Attention

Over the past 7 days, if your child appeared tired, how many days was it challenging for them to pay attention either at school or at home?

- ◯ 0
- ◯ 1
- ◯ 2
- ◯ 3
- ◯ 4
- ◯ 5
- ◯ 6 or more

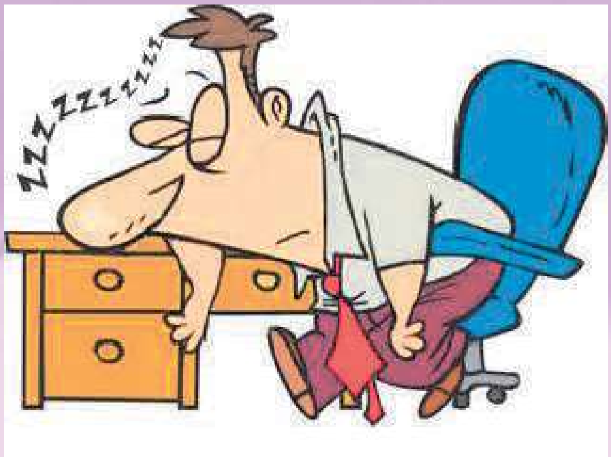

Thoughts of Tiredness over the Past 7 Days (Child)

Please select a choice for each of the questions below about how you felt if you were tired any day over the past 7 days

Study ID #: (Provided by Study Team)

Tiredness Over the Past 7 Days

Today's Date

Month Day Year

Fatigue & Spending Time with Friends

If you felt tired over the past 7 days, how many days was it hard for you to go outside, play with friends, or do any other activities that your enjoy?

- 0
- 1
- 2
- 3
- 4
- 5
- 6 or more

QuestionPro

Fatigue & Starting & Completing Activities

If you were tired over the past 7 days, how many days was it hard for you to start or finish activities that you usually do every day?

- ◯ 0
- ◯ 1
- ◯ 2
- ◯ 3
- ◯ 4
- ◯ 5
- ◯ 6 or more

Fatigue & Schoolwork

If you were tired over the past 7 days, how many days was it hard for you to do your schoolwork?

- ◯ 0
- ◯ 1
- ◯ 2
- ◯ 3
- ◯ 4
- ◯ 5
- ◯ 6 or more

Fatigue & Paying Attention

If you were tired over the past 7 days, how many days was it hard for you to pay attention either at school or at home?

- ○ 0
- ○ 1
- ○ 2
- ○ 3
- ○ 4
- ○ 5
- ○ 6 or more

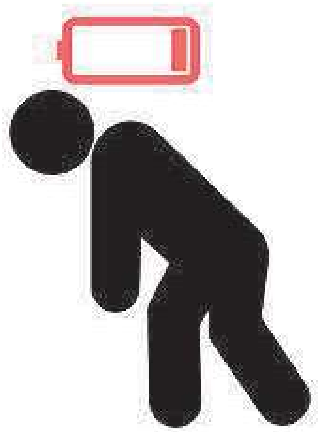

Child's Daily Thoughts of Tiredness (Parent/Guardian)

Please select a choice for each of the questions below about how your child felt if they were tired today

Study ID #: (Provided by Research Team)

Daily Thoughts of Child's Tiredness

Today's Date

Month Day Year

Fatigue & Enjoyment

If your child appeared tired today, was it difficult for them to go outside, play with friends, or do anything they typically enjoy doing as much as they would like?

- ◯ Yes
- ◯ No
- ◯ NA-- my child did not appear tired

Fatigue & Starting & Completing Activities

If your child appeared tired today, was it difficult for them to start or complete activities that are normally done every day?

◯ Yes

◯ No

◯ N/A-- my child did not appear tired

Fatigue & Schoolwork

If your child appeared tired today, was it challenging for them to keep up with their schoolwork?

◯ Yes

◯ No

◯ N/A-- my child did not appear tired

Fatigue & Paying Attention

If your child appeared tired today, did you notice that it was challenging for them to pay attention either at school or at home?

◯ Yes

◯ No

◯ N/A-- my child did not appear tired

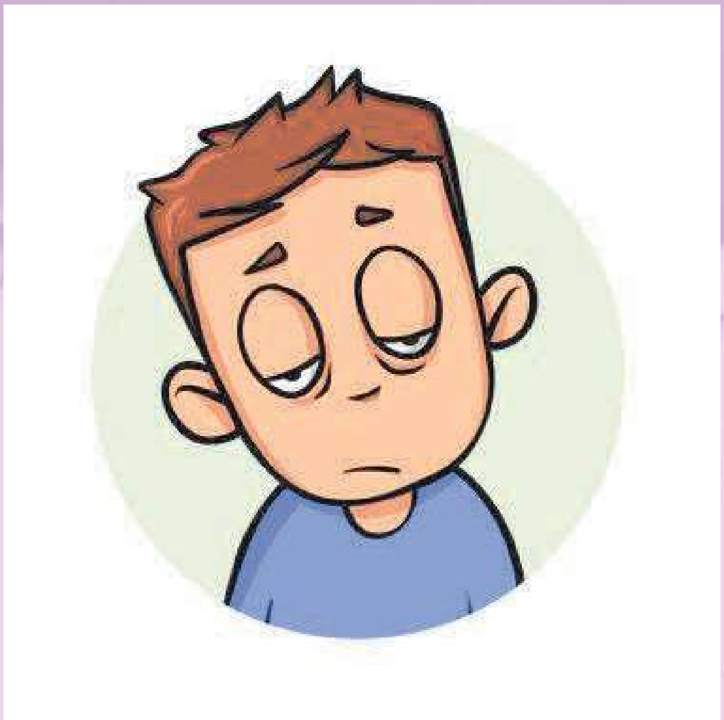

Daily Thoughts of Tiredness (Child)

Please select choose a choice that best describes you during the day when you're feeling tired

Study ID #: (Provided by Research Team)

Daily Thoughts of Tiredness

Today's Date

Month | Day | Year

Fatigue & Enjoyment

If you were tired today, was it hard for you to go outside, play with friends, or do other things you enjoy?

- Yes
- No
- NA-- I was not tired today

Fatigue & Starting & Completing Activities

If you were tired today, was it hard for you to start or finish activities that you usually do every day?

QuestionPro

- ◯ Yes
- ◯ No
- ◯ N/A-- I was not tired today

Fatigue & Schoolwork

If you were tired today, was it hard for you to do your schoolwork?

- ◯ Yes
- ◯ No
- ◯ N/A-- I was not sleepy today

Fatigue & Paying Attention

If you were tired today, was it hard for you to pay attention either at school or at home?

- ◯ Yes
- ◯ No
- ◯ N/A-- I was not tired
